# Combination of sunitinib and ^177^Lu-labeled antibody cG250 targeted radioimmunotherapy: A promising new therapeutic strategy for patients with advanced renal cell cancer

**DOI:** 10.1016/j.neo.2022.100826

**Published:** 2022-07-22

**Authors:** Jeannette C. Oosterwijk-Wakka, Mirjam C.A. de Weijert, Gerben M. Franssen, Dimitar R. Kolev, Ton A.F.J. de Haan, Otto C. Boerman, Peter F.A. Mulders, Egbert Oosterwijk

**Affiliations:** a Radboud university medical center, Department of Urology, 267 Experimental Urology, PO Box 9101, 6500 HB Nijmegen, the Netherlands; b Radboud university medical center, Department of Radiology and Nuclear Medicine, PO Box 9101, 6500 HB Nijmegen, the Netherlands; c Radboud university medical center, Department for Health Evidence, PO Box 9101, 6500 HB Nijmegen, the Netherlands; d Radboud university medical center, Animal research facility, PO Box 9101, 6500 HB Nijmegen, the Netherlands; e Radboud university medical center, Department of Urology, PO Box 9101, 6500 HB Nijmegen, the Netherlands

**Keywords:** Sunitinib, RCC, Combination therapy, CAIX-targeted radioimmunotherapy, [^177^Lu]Lu-cG250 RIT, Sunitinib resistance, mRCC, metastatic renal cell carcinoma, ccRCC, clear cell renal cell carcinoma, RIT, radioimmunotherapy, IC, immune checkpoint, CAIX, carbonic anhydrase 9, MVD, microvessel density, Su, sunitinib

## Abstract

Sunitinib is an effective treatment for patients with metastatic Renal Cell Carcinoma (mRCC) but ultimately resistance occurs. The aim of this study was to investigate sunitinib resistance in RCCs and to develop therapeutic combination strategies with targeted radioimmunotherapy (RIT).

We studied two RCC models, analyzed Vascular endothelial growth factor (VEGF) and its receptor (VEGFR) and AXL/MET expression and performed therapy studies in Balb/c^nu/nu^ mice combining sunitinib and [^177^Lu]Lu-cG250 RIT (6.5 MBq/10 μg), specifically targeting RCC cells.

pAXL and pMET were expressed in sunitinib-resistant SK-RC-52 and absent in sunitinib-sensitive NU12. NGS evaluation showed that expression of VEGFA, VEGFB, VEGFD, PGF and VEGFR1,2,3 was higher and expression of VEGFC and PDGFA was lower in NU12 than in SK-RC-52.

Therapy studies combining sunitinib with [^177^Lu]Lu-cG250 RIT showed that the best response in mice with “resistant” SK-RC-52 tumors was observed with two cycles of Sunitinib and ^[177^Lu]Lu-cG250 RIT, probably due to increased vascular permeability by sunitinib treatment. In the “sensitive” NU12 model, two cycles of [^177^Lu]Lu-cG250 RIT and two cycles of combination treatment were equally effective.

Enhanced therapeutic efficacy was achieved when two agents ([^177^Lu]Lu-cG250 RIT and sunitinib) that on their own did not induce satisfactory response levels, are combined. Our findings provide a promising new therapeutic strategy for patients with advanced RCC.

## Introduction

The last two decades substantial progress has been made in understanding the underlying molecular mechanisms of clear cell renal cell carcinoma (ccRCC) leading to significant number of new treatment options for patients with metastatic RCC (mRCC), comprising of Tyrosine kinase inhibitors (TKI) and immune checkpoint (IC) inhibitors [1], [2], [3]. Implementation of TKI in 2007 has improved objective response rates (ORR) and median progression free survival (PFS) substantially [4,5].

Since RCC is considered a highly immunogenic tumor, IC-based therapies were also developed either as monotherapy or in combination: pembrolizumab/axitinib, nivolumab/cabozantinib, pembrolizumab/ Lenvatinib and nivolumab/ipilimumab are now approved as first line treatment of patients with mRCC [6,7]. However, for patients who cannot receive or do not tolerate IC inhibitors, monotherapy with sunitinib, pazopanib and cabozantinib is still the preferred first-line treatment. Moreover, after IC failure any TKI that has not been used in combination with an IC is standard of care. Thus, TKI still play a major role in the clinical management of mRCC patients.

Previously we studied the effect of sunitinib treatment on antibody targeting in two RCC models, SK-RC-52, a sunitinib-resistant model and NU12, a sunitinib-sensitive model to investigate the possibility of combination therapy [8], for patients developing therapy-resistance to TKI. Activation of alternative signaling pathways like AXL and MET plays an important role in the development of resistance: it leads to angiogenesis, tumor survival, invasion, and metastasis [9,10]. Combination of two treatment modalities with a different working mechanism might lead to improved outcome.

Since the sunitinib target, i.e., the vascular component, is of host origin, we expected a similar effect of sunitinib in both models. However, depending on the model studied, sunitinib treatment resulted in extensive necrosis and decreased microvessel density (MVD) (NU12) or minimal tumor necrosis and unchanged MVD (SK-RC-52).

Parallel to the different sunitinib response the models differed substantially in the accumulation of cG250, a monoclonal antibody targeting Carbonic anhydrase IX (CAIX) which is highly expressed in clear cell RCC (ccRCC) [11]. Sunitinib treatment significantly decreased cG250 accumulation in NU12 but the reverse, increased tumor accumulation was seen in SK-RC-52. How this relates to therapeutic effects of CAIX-targeted radioimmunotherapy is unknown. This is relevant because we have previously demonstrated therapeutic responses in mRCC patients in a clinical study of single-agent CAIX-targeted RIT with

[^177^Lu]Lu-DOTA-cG250, albeit that the responses were limited [12]. Altered pharmacokinetic and pharmacodynamic behavior will certainly influence therapeutic outcome.

In this study we aimed to explain the observed differences of SK-RC-52 and NU-12 in response to sunitinib and studied whether we could develop a therapeutic combination strategy aiming at the tumor vasculature and tumor cells amendable to both sunitinib-sensitive and sunitinib-resistant RCC.

## Material and methods

### Cell lines, xenografts and reagents

The human RCC cell line SK-RC-52 was established from a mediastinal metastasis of a primary RCC [27]. NU12 was initially maintained as a xenograft established from a primary tumor of a metastasis RCC patient [28]. Cell line NU12 was a derivative of the NU12 xenograft. For NU12 cell culture, culture plastics were coated with FNC-coating mix (Enzo life sciences) before use. Cells were maintained in RPMI1640 (Gibco, Bleiswijk, The Netherlands) supplemented with 10% fetal bovine serum (Sigma-Aldrich, Zwijndrecht, The Netherlands) and 2 mM glutamine (Gibco). Human RCC xenograft model NU12 [28] was maintained by passing freshly excised tumor pieces (1-2 mm^3^) subcutaneously (s.c.) in mice. Both SK-RC-52 and NU12 express high levels of CAIX [26]. SK-RC-52 was authenticated by Eurofins. NU-12 was not present in the online DSZB database.

Sunitinib-resistant cells were obtained by weaning cells to escalating concentrations from 4 up to 10 µM sunitinib and subsequently by continuous culturing at 10 µM of sunitinib (SuR cells). Medium containing sunitinib was refreshed every 2-3 days. Sunitinib (Selleckchem S1042, Bioconnect, Huissen, The Netherlands) was prepared fresh from stock solution (20 mM in DMSO).

### Analysis of cell viability and morphology

NU12 or SK-RC-52 cells were harvested and seeded in tissue culture treated 96-well plates (Costar 3596, VWR, Amsterdam, The Netherlands) at 2000 cells/well in 100 µl in triplicate. Twenty-four hours after seeding, cells were treated with 0.02- 20 µM sunitinib or N-desethyl sunitinib (Toronto chemicals D289650, North York, Canada), freshly prepared from 20mM stocks in DMSO. Final concentration DMSO in all wells was 0.2%. Cell viability was measured 72 h after start of treatment with CellTiter-Glo (Promega, Leiden, The Netherlands) according to manufacturer's instructions and luminescent signal was analyzed with a Victor3 1420 Multi Label Counter (Perkin Elmer, Groningen, The Netherlands). Average luminescent signal was depicted as % viable cells of control.

The effect of sunitinib treatment was also analyzed by live imaging (IncucyteZoom live cell imaging system, Essen Bioscience, Sartorius, Royston, UK). Confluence of untreated cells was measured for 24 h. Then cells were treated with 0.16-40 μM of sunitinib for 72 h and confluence was measured every 2 h. IncucyteZoom software (2016B) was used to analyze the data obtained. Doubling times for untreated cells in their exponential growth phase were calculated using the Doubling Time Software (Roth V. 2006, http://www.doubling-time.com/compute_more.php). Data are presented as mean ± standard deviation (SD) for growth curves and doubling times.

### Western blot analysis

Cells of SK-RC-52, NU12 and their sunitinib resistant derivatives (SuR) were cultured to 100% confluency, washed with 0.9% NaCl and lysed with Laemmli buffer (60 mM Tris glycine pH 6.8, 1 mM CaCl_2_, 2 % SDS and 355 mM 2-mercaptoethanol). Cell lysates were separated on 7.5 % SDS-PAGE gels and transferred to polyvinylidene fluoride (PVDF) membranes (Amersham Hybond, VWR). Blots were washed with 20mM Tris-buffered saline (TBS) supplemented with 0.001 % Tween-20 (TBST) and incubated in TBST with 5 % bovine serum albumin (BSA, Sigma-Aldrich) (blocking buffer) overnight at 4°C.

After washing, membranes were incubated either for 2 h at room temperature (RT) or overnight at 4°C with primary antibodies: c-MET (#4560, 1:1000), p-MET Tyr1234/35 (clone D26, # 3077, 1:500), both cell signaling, Leiden, the Netherlands, p-Met Tyr1349 (clone EP2367Y, Abcam, Cambridge, UK, 1:1000), AXL (#13196-1-AP, Proteintech, Bioconnect, Huissen, The Netherlands 1:1000), p-AXL Tyr702 (clone D12B2, #5724, cell signaling, 1:100) and β-actin (#A5441, Sigma-Aldrich,1:5000) diluted in blocking buffer. Subsequently, blots were washed and incubated for 1 h at RT with fluorescent-dye conjugated secondary antibodies (Goat anti-rabbit IgG (H+L) Alexa Fluor 680, #A-21076; Goat anti-mouse IgG (H&L), DyLight 800 4X PEG, #SA5-35521, 1:5000) (both Thermo Fisher Scientific, Eindhoven, The Netherlands) in blocking buffer.

After a final washing step, blots were scanned with the Odyssey CLx imaging system and protein bands were quantified using the Image Studio Lite Version 5.2 software (both LI-COR Biosciences, Miami, USA). Following background correction and normalization of target protein levels to endogenous β-actin content, each experimental condition was compared to untreated cells.

### RNA-Seq analysis

SK-RC-52 cells, NU12 cells and xenografts harvested from untreated Balb/c nu/nu mice were profiled using RNA sequencing (Illumina). In short, libraries were generated from RNA starting material and paired-end library sequencing was performed with NextSeq500 Illumina platform.

Expression levels were normalized using HPRT gene expression as standard. To evaluate gene expression levels of the mouse endothelium of xenografts, the primary target of sunitinib, mouse gene expression was separated from human gene expression as described [29]. Data Availability Statement: The dataset used in this study is available in EGA (https://ega-archive.org) with access number EGAD00001008312.

ENSG00000197461 (PDGFA), ENSG00000100311 (PDGFB), ENSG00000112715 (VEGFA), ENSG00000173511 (VEGFB), ENSG00000150630 (VEGFC), ENSG00000165197 (VEGFD), ENSG00000119630 (PGF), ENSG00000134853 (PDGFRA), ENSG00000113721 (PDGFRB), ENSG00000102755 (FLT1/VEGFR1), ENSG00000128052 (KDR/VEGFR2), ENSG00000037280 (FLT4/VEGFR3).

### Conjugation and radiolabeling of cG250

The conjugation of cG250 (generously provided by Wilex AG, Munich, Germany) to isothiocyanato-benzyl-1,4,7,10-tetraazacyclododecane-1,4,7,10-tetraacetic acid (ITC-DOTA) was performed essentially as described by Lewis et al. [30]. In brief, cG250 was conjugated with ITC-DOTA (Macrocyclics, Dallas, TX) in 0.1 M NaHCO_3,_ pH 9.5 for 1 h at RT, using a 15-fold molar excess of ITC-DOTA. To remove unbound ITC-DOTA, the reaction mixture was dialyzed extensively against 0.25 M ammoniumacetate buffer, pH 5.5 containing chelex 100 resin 2 g/L.

The cG250-ITC-DOTA conjugate (150-350 µg) was radiolabeled with 200-450 MBq ^177^Lu, no-carrier-added (ITM medical isotopes) in 0.5 MES buffer, pH 5.5 for 20 min at RT under strict metal-free conditions. After incubation, 50 mM EDTA was added to a final concentration of 5 mM [13].

Instant Thin Layer Chromatography (ITLC) was used to determine labeling efficiency of the [^177^Lu]Lu -cG250 preparations using silica gel strips (Agilent technologies, Amstelveen, The Netherlands) and 0.1 M citrate buffer pH 6.0 as the mobile phase. When labeling efficiency was < 95%, the reaction mixture was purified on a PD-10 column (GE). The radiochemical purity exceeded 95% in all experiments. The immunoreactive fraction (IRF), determined on freshly harvested SK-RC-52 RCC cells at infinite antigen excess essentially as described by Lindmo et al. [31] with minor modifications [8], exceeded 75% in all experiments.

### In vivo therapy experiments

Institutional guidelines were strictly followed for maintenance of animals and experimental procedures were approved by the Institutional Animal Care and Use Committee (IACUC, RU-DEC 2012-038 and RU-DEC 2012-267). All procedures were performed using the guidelines from the Institute of Laboratory Animal Research [32]. Female BALB/c nu/nu mice, 6-8 weeks of age, were obtained from Janvier, France, and maintained at the local central animal facility. Animals were either injected s.c. with 2*10^6^ freshly harvested SK-RC-52 cells or grafted s.c. with freshly excised NU12 xenograft pieces of approximately 1-2 mm^3^ .

Mice were randomly divided into groups of 10-14 mice once tumors reached the desired volume (50-150 mm^3^) and treatment was initiated. Included number of mice with SK-RC-52 tumors was: 13, 10, 11, 12, 13 and 10 for Su+^177^Lu-cG250 RIT one cycle, Su+^177^Lu-cG250 RIT 2 cycles, ^177^Lu-cG250 RIT one cycle, ^177^Lu-cG250 RIT two cycles, Su two cycles, and Control respectively. Included number of mice was 12, 13, 14, 14, 14 and 14 for Su+^177^Lu-cG250 RIT one cycle, Su+^177^Lu-cG250 RIT two cycles, ^177^Lu-cG250 RIT one cycle, ^177^Lu-cG250 RIT two cycles, Su two cycles, and control respectively.

In Fig. S1, the treatment schedule is illustrated for mice with SK-RC-52 and NU12 tumors. Mice received the equivalent of 40 mg/kg (0.8 mg/200 µl) Sunitinib (SU11248, Sutent®, Pfizer) dissolved in 0.1M Na citrate, pH 4.5 orally per day for 14 days. Three days thereafter, mice received 6.5 MBq/10 μg [^177^Lu]Lu-cG250 (1/3 of Maximum Tolerated Dose) by intravenous injection (1st cycle). Six (NU12) or 7 (SK-RC-52) weeks after start of the 1^st^ cycle another treatment cycle was administered (Su + [^177^Lu]Lu-cG250 RIT 2x). Comparator groups were treated with one cycle of combined treatment (Su + [^177^Lu]Lu-cG250 RIT 1x), 1 or 2 cycles of [^177^Lu]Lu -cG250 RIT ([^177^Lu]Lu -cG250 1x/ 2x), two cycles of sunitinib (Su 2x) or were left untreated (control). Tumor volumes were determined twice a week by caliper measurements by an evaluator blinded to the treatment groups. Tumor volume was estimated using the following formula: (length x width x depth) x π/6. The last observed tumor volume was used to calculate average tumor volumes, i.e., when mice were euthanized.

Mice were euthanized when either tumor burden reached 1500 mm^3^ or when mice reached a predetermined humane endpoint. After the animals were euthanized, tumors were dissected and analyzed. Kaplan-Meier curves for overall survival (OS) were generated. Mice that were sacrificed because of other reasons than reaching maximal tumor burden were excluded for analysis.

### Immunohistochemical analysis

After euthanizing mice, tumors were harvested, embedded in tissue-tek® O.C.T. compound (Sakura, Alphen a/d Rijn, The Netherlands), snap-frozen in dry ice cooled isopentane and stored at -80°C and/or formalin-fixed and paraffin embedded. Four µm cryostat sections were stored at -80°C until use. morphological analysis of the tumors was performed by Hematoxylin-Eosin (HE) staining.

Primary antibodies used were chimeric mAb anti-human CAIX (mAb cG250, Wilex, 10 µg/mL), rabbit-anti-human mAb Ki67 (clone sp6/RM-9106-S, Thermo Fisher Scientific, 1:200), rabbit-anti-human mAb MET (D1C2, cell signaling #8198S, 1:200), polyclonal rabbit-anti-human AXL (Proteintech #13196-1-AP, 1:250).

For visualization of cell proliferation, paraffin sections were deparaffinized and rehydrated. Endogenous peroxidase was blocked with 3% H_2_0_2_ in PBS for 5 min. Slides were washed with PBS and antigen retrieval was performed in 0.1 M citrate buffer, pH 6.0 for 10 min. Subsequently sections were blocked with 20% normal swine serum and incubated with primary antibody Ki67 diluted in 1% BSA/ PBS. For visualization of MET, AXL and CAIX expression, frozen sections were fixed in acetone, washed and incubated with primary antibody. After washing, sections were incubated with PO conjugated swine-anti-rabbit IgG (Dako, Amstelveen, The Netherlands), 1:100, pre-incubated with 4% normal mouse serum. All sections were developed with bright DAB (Immunologic, Duiven, The Netherlands) and counterstained with hematoxylin.

Microscopic evaluation was performed on an Axioskop microscope (Zeiss, Breda, The Netherlands) and images acquired on the Axiocam mrc5 with Axio vs40 version 4.8 2.0 software (Axiovision, Zeiss).

### Statistical analysis

A mixed model analysis was used to compare the tumor growth in the six treatment groups. For each treatment group the development in time was described with a fifth-degree polynomial (fixed effects). The individual mice were allowed to follow their own curve (all six coefficients for the polynomial function in time were random). Using this model, the geometric mean tumor volume at the end of sunitinib 1^st^ cycle (Day 14), start of 2^nd^ cycle (day 42 or 48), end of sunitinib 2^nd^ cycle (day 55/62), evaluation of 2^nd^ cycle (day 91/98 and end of experiment (day147/160) for NU12/SK-RC-52 respectively) was compared between treatment groups for selected hypotheses. Correction for multiple comparisons was done per day using Holm's method (improved Bonferroni method) [33].

For survival analysis, p-values were calculated from the log rank or Gehan-Breslow-Wilcoxon test corrected for multiple comparisons. p<0.05 was considered significant.

## Results

Previously we studied the effect of TKI treatment on the accumulation of CAIX‐specific chimeric monoclonal antibody cG250 in two RCC mouse models, NU12 and SK-RC-52, and noticed that both models reacted differently to sunitinib treatment [8]. NU12 behaved as a sunitinib ‘sensitive’ tumor: *in vivo* sunitinib treatment led to massive necrosis and decreased MVD and concomitantly accumulation of monoclonal antibody cG250, specific for CAIX which is highly expressed on ccRCC, was markedly reduced. In contrast, in SK-RC-52 tumors necrosis was hardly observed, MVD remained unchanged and accumulation of cG250 increased after sunitinib treatment. Because the vascular component of the xenografts is of the same host origin, we considered whether intrinsic differences between the tumor cells could explain our observations.

### Viability of NU12 and SK-RC-52 treated with sunitinib

**Fig. 1:**
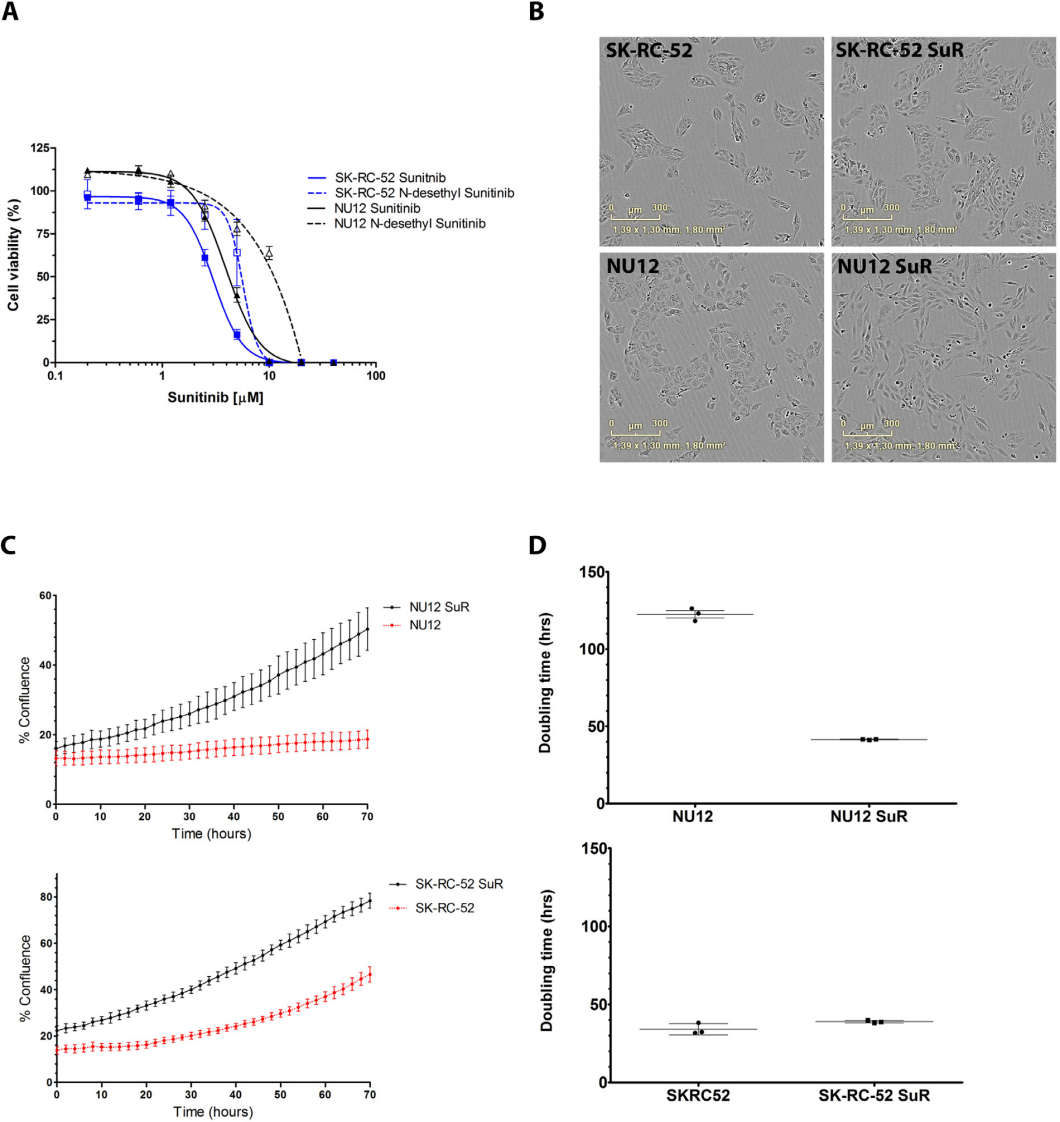
Cell growth and expression of vascular endothelial growth factor (VEGF) or vascular endothelial growth factor receptor (VEGFR) in NU12 and SK-RC-52 treated with sunitinib**. (A)** Cell viability of NU12 (black curves) and SK-RC-52 (blue curves) treated for three days with increasing concentrations of sunitinib (solid lines) or N-desethyl-sunitinib (dotted curve). (**B)** Morphology of NU12, Sunitinib-resistant (SuR) NU12 (NU12 SuR), SK-RC-52 and SK-RC-52 SuR cells. Phase-contrast images were obtained 72 h after seeding. (**C)** Tumor cells of NU12, NU12 SuR, SK-RC-52 or SK-RC-52 SuR were seeded (2000 cells/well) in triplicate in a 96-wells plate and cell growth was monitored for three days in IncucyteZoom system. NU12 (top), SK-RC-52 (bottom), untreated cells (red lines), SuR (black lines). (**D)** Doubling times of NU12, NU12 SuR, SK-RC-52 and SK-RC-52 cells.

Dose-response analysis showed that the viability of NU12 and SK-RC-52 cells decreased significantly from doses of 2.5 μM sunitinib and beyond. Cells did not survive treatment with 10 μM sunitinib for 7 days (Fig. 1A). IC_50_ values of the cell lines were 3.9 μM and 3 μM sunitinib for NU12 and SK-RC-52, respectively. Dose-response analysis with the active form N-desethyl sunitinib showed that the IC_50_ values were 10.5 μM and 5.6 μM for NU12 and SK-RC-52 cells respectively.

### Cell growth and morphology

Since the IC_50_ values did not differ substantially, it was unlikely that this could explain the difference in NU12 and SK-RC-52 sunitinib sensitivity *in vivo.* We examined the morphology of parental and Sunitinib-resistant (SuR) cells and did not observe morphological changes of SK-RC-52 after long term treatment with 10 μM Sunitinib, but the morphology of the NU12 cells changed from a cuboidal shape to a more fibroblastic, spindle type morphology, possibly indicating a more aggressive cell type (Fig. 1B).

Next, we investigated cell growth of the parental and SuR cells with life cell imaging. Growth speed of NU12 SuR cells was enhanced compared to the parental cells (Fig. 1C). Confluence of NU12 increased from approximately 13% to 19% in three days of culture and NU12 SuR confluence increased from 16% to 52%. (Fig. 1C, top). In agreement, doubling time of NU12 SuR cells was substantially shorter: 42 h ± 0.3 *versus* 122 h ± 4 for the parental NU12 cells (Fig. 1D). In contrast, for SK-RC-52 the difference in growth speed between untreated and SuR cells was much smaller: Confluence of SK-RC-52 and SK-RC-52 SuR increased from 14% to 47% and 22% to 78% respectively (Fig. 1C, bottom), and the doubling time was similar: 34±4 h and 39±0.9 h for SK-RC-52 and SK-RC-52 SuR, respectively.

### VEGF and VEGFR expression by RNA-Seq analysis of NU12 and SK-RC-52 xenografts and cells

To investigate whether the differences between the different *in vivo* responses could be explained by VEGF and VEGFR gene expression levels, we performed RNA sequencing on NU12 and SK-RC-52 cells and xenografts (Table 1). After correction using HPRT gene expression as standard, NU12 VEGFA, VEGF-B and VEGF-D levels were substantially higher (ratio of 4.8, 2.7 and 1.8 respectively) and VEGFC and PDGFA levels were lower (ratio of 0.06 and 0.5 respectively) compared to SK-RC-52 cells.

**Table 1:** Gene expression levels of NU12 and SK-RC-52.

NU12/SK-RC-52 ratio			
Gene	tumor cells	Gene	mouse endothelium
ENSG00000197461| PDGFA	0.57	ENSG00000134853| PDGFRA	0.8
ENSG00000100311| PDGFB	0.79	ENSG00000113721| PDGFRB	1.15
ENSG00000112715| VEGFA	0.96	ENSG00000102755| FLT1 VEGFR1	2.37
ENSG00000173511| VEGFB	0.87	ENSG00000128052| KDR VEGFR2	1.27
ENSG00000150630| VEGFC	0.53	ENSG00000037280| FLT4 VEGFR3	-
ENSG00000165197| VEGFD	2.06		

Gene expression levels were generated with RNA sequencing of NU12 tumor cells, SK-RC-52 tumor cells, mouse endothelium of NU12 or SK-RC-52 xenografts. Shown are ratios of NU12:SK-RC-52 of relevant vascular endothelial growth factor (VEGF)/ vascular endothelial growth factor receptor (VEGFR) genes. PGF: Placental growth factor.

To evaluate gene expression levels of the mouse endothelium of xenografts, the primary target of sunitinib, mouse gene expression was separated from human gene expression showing that in endothelium of NU12 xenografts, expression levels of VEGFR1-2, the most important sunitinib targets, were higher compared to SK-RC-52 (NU12: SK-RC-52 ratio of 11.3 and 3.5 respectively) while VEGFR3 was expressed in NU12 only. Extraordinary high levels of PGF were detected in NU12 tumorcells (NU12/SK-RC-52 ratio of 271).

### MET and AXL expression in NU12 or SK-RC-52 xenografts of mice treated with sunitinib

**Fig. 2:**
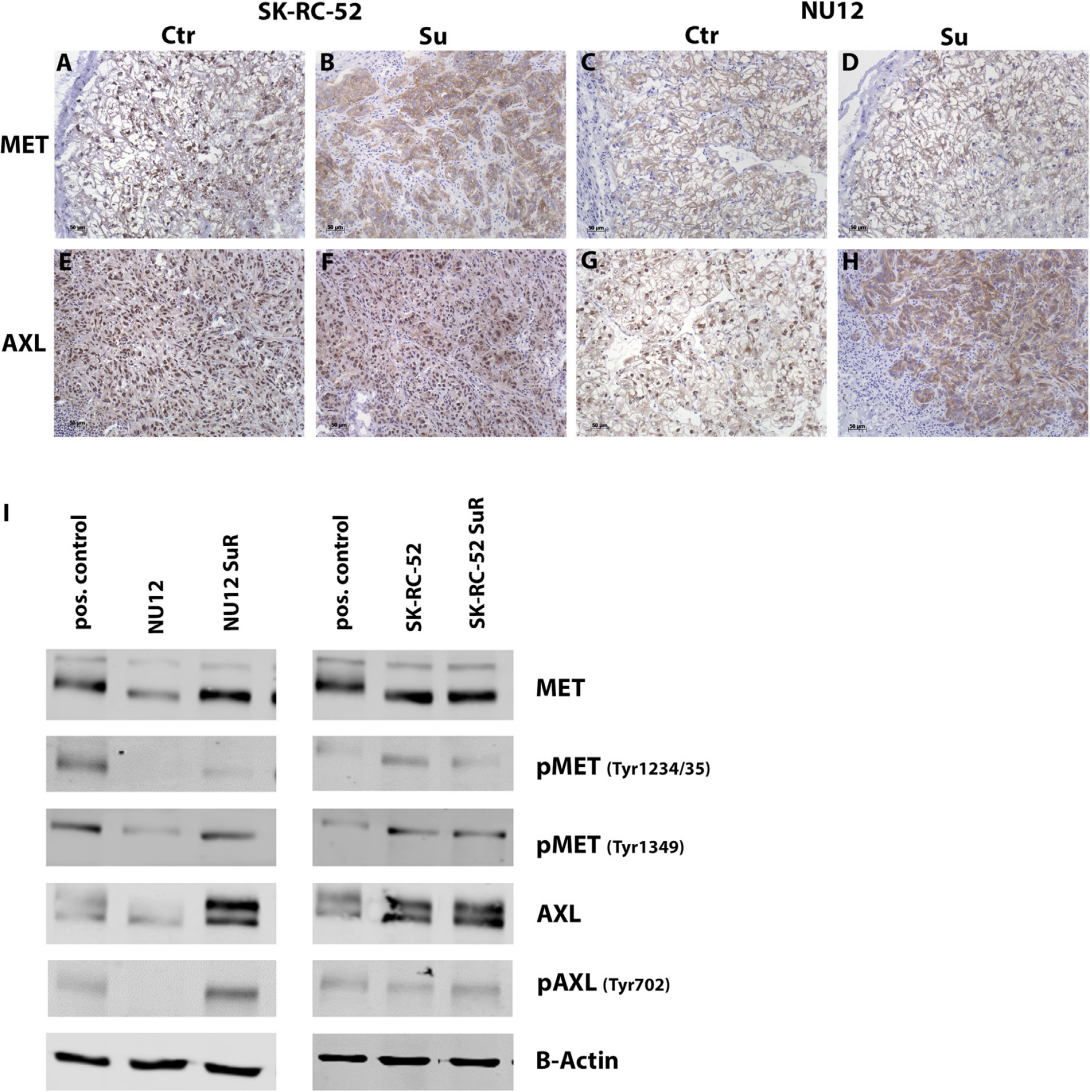
MET and AXL expression of NU12 and SK-RC-52 treated with sunitinib. (**A-D)** MET expression in xenografts, (**E-H)** AXL expression in xenografts, (**A/E)** SK-RC-52 xenografts of untreated mice (ctr), (**B/F)** SK-RC-52 xenografts of sunitinib treated mice (Su), (**C/G)** NU12 xenografts of untreated mice (ctr), (**D/H)** NU12 xenografts of sunitinib treated mice (Su), **I**: Western blot analysis of (p)MET and (p)AXL expression in NU12, sunitinib resistant (SuR) NU12 (NU12 SuR), SK-RC-52, SK-RC-52 SuR tumor cells. β-actin was used as loading control. Representative images were obtained with Odyssey CLx.

Because the observed differences might also be the consequence of altered and/or constitutive activation of cell signaling pathways, we tested (p)MET and (p)AXL expression and found that MET was abundantly expressed in SK-RC-52 xenografts, regardless of sunitinib treatment (Fig. 2A-B), Likewise, MET expression was unchanged in sunitinib-treated NU12 xenografts compared to untreated tumors (Fig. 2C-D). Also, NU12 and SK-RC-52 AXL expression was not influenced by sunitinib treatment (Fig. 2E-F, and 2G-H).

Long-term sunitinib treatment of NU12 cells resulted in strong upregulation of MET and AXL (Fig. 2I and Fig. S2). Additionally, NU12 SuR cells clearly expressed pMET and pAXL, in contrast to NU12 which was pAXL negative and pMET negative (pMET(tyr1234/35)) or weakly positive (pMET(tyr1349)).

Long-term sunitinib treatment of SKRC52 did not alter the high MET and AXL expression (Fig. 2I and Fig. S3). Both pMET and pAXL were present in SK-RC-52 and SK-RC-52 SuR cells, implying constitutive active pathways, regardless of sunitinib treatment.

### In vivo experiments

To exploit differences in sunitinib response of NU12 and SK-RC-52 tumors, xenografted BALB/c nu/nu mice were treated with one or two cycles of sunitinib combined with [^177^Lu]Lu -cG250 RIT to examine whether the therapy response differed in these two models. To be able to evaluate the additive or synergistic effect of the individual components of the combination therapy, the ^177^Lu activity dose was reduced to one-third of the maximum tolerated dose that was previously described for SK-RC-52 [13].

### Treatment of mice with SK-RC-52 tumors

**Fig. 3:**
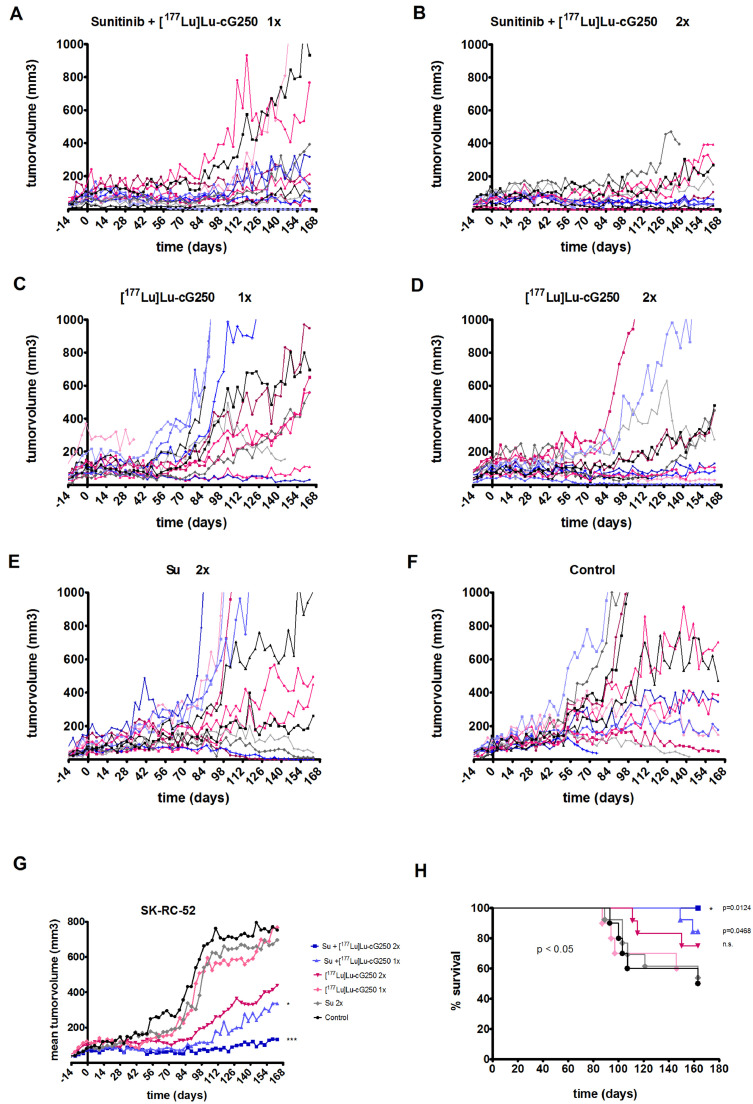
Tumor growth of SK-RC-52 tumors and survival of mice during treatment with sunitinib (Su) and/or [^177^Lu]Lu -cG250 radioimmunotherapy (RIT). (**A-F)** Growth curves of individual mice. (**A)** Su+[^177^Lu]Lu -cG250 RIT one cycle, (**B)** Su+[^177^Lu]Lu -cG250 RIT two cycles, (**C)** [^177^Lu]Lu -cG250 RIT one cycle, (**D)** [^177^Lu]Lu -cG250 RIT two cycles, (**E)** Su two cycles, (**F**) Control. (**G)** Mean tumor volume of all treatment groups, * p < 0.05, *** p < 0.001. P-values shown are Holm's adjusted for the comparison of mean tumor volumes for comparisons of all treatment groups vs control group on day 98 (end of 2^nd^ cycle). (**H)** overall survival of treatment groups. Mice that were sacrificed because of other reasons than reaching maximal tumor burden, were excluded. Overall comparison of survival curves was significant with p<0.05. P-values shown for the Su+[^177^Lu]Lu -cG250 RIT one cycle, two cycles and [^177^Lu]Lu -cG250 RIT two cycles all vs Control were calculated from the log rank or Gehan-Breslow-Wilcoxon test corrected for multiple comparisons (p=0.0124 for Su+[^177^Lu]Lu -cG250 RIT two cycles vs Control, p= 0.0468 for Su+[^177^Lu]Lu -cG250 RIT one cycle vs Control and p= 0.129 for [^177^Lu]Lu -cG250 RIT two cycles vs Control).

Treatment was started approximately 4 weeks after tumor cell inoculation, when tumors reached 80 mm^3^ ± 35 mm^3^. No significant delay of SK-RC-52 tumor growth was observed after treatment with one cycle of sunitinib (Su) (Fig. 3E,G); p= 0.168, p= 0.442 at day 14 and day 48 respectively) or one cycle of [^177^Lu]Lu -G250 (Fig. 3C p=0.126 at day 48). In contrast, one cycle of Su + [^177^Lu]Lu-cG250 RIT resulted in a significant long-lasting tumor growth delay (Fig. 3A,G; Table 2; Table S2, p< 0.001 day 48).Two cycles of [^177^Lu]Lu -cG250 RIT resulted in a moderate, but not significant tumorgrowth delay of SK-RC-52 tumors (Fig. 3D,G p=0.123; day 98). However, two sequential sequences combining sunitinib with [^177^Lu]Lu-cG250 RIT resulted in almost complete tumor stasis (Fig. 3B,G). (p<0.001). Of note, 91% and 85% of mice survived when treated with two cycles or one cycle combination therapy, respectively (Table S1). In contrast, survival was lower in mice treated with [^177^Lu]Lu-cG250 RIT: 69% of mice were alive after two cycles and 54% after one cycle of [^177^Lu]Lu -cG250 RIT. Survival after two cycles of sunitinib treatment was 54% (Fig. 3H and Table S1).

**Table 2:** Median tumor volumes of SK-RC-52.

	Day														
	14			48			62			98			160		
	Vol (mm^3^)	95% confidence interval		Vol (mm^3^)	95% confidence interval		Vol (mm^3^)	95% confidence interval		Vol (mm^3^)	95% confidence interval		Vol (mm^3^)	95% confidence interval	
Control	**102**	73	142	**164**	104	258	**201**	120	336	**323**	145	720	**311**	68	1418
Su,2x	**88**	63	123	**127**	80	201	**152**	90	254	**261**	117	580	**279**	63	1230
Lu-cG250 1x	**110**	78	153	**101**	64	160	**122**	73	206	**314**	138	714	**574**	123	2672
Lu-cG250 2x	**103**	74	144	**92**	58	145	**87**	52	145	**93**	42	206	**207**	49	880
Su + Lu-cG250 1x	**73**	52	102	**58**	36	91	**52**	31	87	**58**	26	128	**136**	33	554
Su + Lu-cG250 2x	**59**	42	82	**43**	27	67	**35**	21	59	**27**	12	60	**41**	9	178

Median tumor volumes of SK-RC-52 tumors per treatment group and follow up day with uncorrected 95% confidence limits. Mixed model analysis was used to compare the tumor growth of the six treatment groups. Using this model, the median tumor volume at days 14 (end 1^st^ cycle of sunitinib (Su), 48 (start 2^nd^ cycle), 62 (end 2^nd^ cycle of Su), 98 (evaluation 2^nd^ cycle) and 160 was compared among treatment groups for selected hypotheses. Correction for multiple comparisons was done per day using Holm's method (improved Bonferroni method). Vol: median tumor volume, 1x: one cycle, 2x: two cycles. * p < 0.05, *** p < 0.001 (for calculated p-values see Table S2).

To confirm that animals were completely cured, we examined the absence of viable tumor cells in mice without palpable tumors by HE and Ki67 staining. No viable tumor cells were observed at the end of treatment in two mice treated with two cycles of Su+[^177^Lu]Lu -cG250, one mouse each treated with one cycle of Su+[^177^Lu]Lu-cG250 and one cycle of [^177^Lu]Lu-cG250 and three mice which were treated with two cycles of sunitinib.

### Treatment of mice with NU12 tumors

Treatment was started approximately 17 days after tumor engraftment when NU12 tumor volumes reached 44 mm^3^ ± 27 mm^3^.

**Fig. 4:**
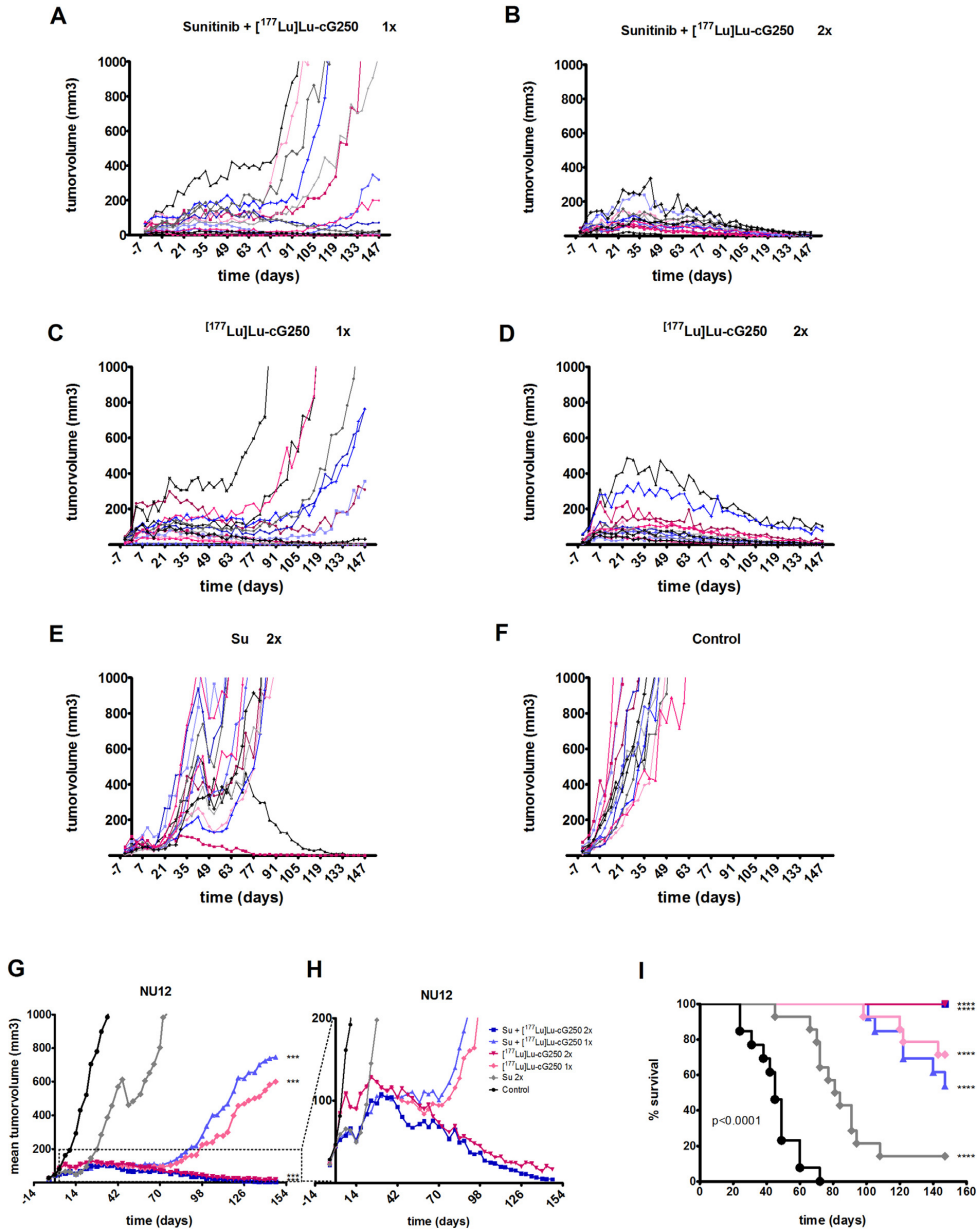
Tumor growth and survival of NU12 tumors during treatment with sunitinib (Su) and/or [^177^Lu]Lu -cG250 radioimmunotherapy (RIT). (**A-F)** Growth curves of individual mice. (**A)** Su+[^177^Lu]Lu cG250 RIT one cycle, (**B)** Su+[^177^Lu]Lu -cG250 RIT two cycles, (**C)** [^177^Lu]Lu -cG250 RIT one cycle, (**D)** [^177^Lu]Lu -cG250 RIT two cycles, (**E)** Su two cycles, (**F)** Control. (**G,H)** Mean tumor volume of all treatment groups. *** P< 0.001. P-values shown are Holm's adjusted for the comparison of mean tumor volumes for comparisons of all treatment groups vs control group on day 91 (end of 2^nd^ cycle) (**I)** Overall survival of treatment groups. Mice that were sacrificed because of other reasons than reaching maximal tumor burden, were excluded. Overall comparison of survival curves was significant with p<0.0001. P-values shown for all treatment groups vs Control were calculated from the log rank or Gehan-Breslow-Wilcoxon test corrected for multiple comparisons (**** p<0.0001). 1x: one cycle. 2x: two cycles.

A significant immediate tumor growth delay was observed in mice treated with one or two cycles of either Su (Fig. 4E), [^177^Lu]Lu -cG250 RIT (Fig. 4C,D) or Su+[^177^Lu]Lu -cG250 RIT (Fig. 4A,B), but substantial differences between the different treatment groups was observed (Fig. 4G,H; Table 3; Table S3). At d14, after one cycle of sunitinib, tumors stabilized (mean tumor volumes ∼60 mm^3^ *versus* 261 mm^3^ control) but rapidly progressed after cessation of treatment (tumor volume d42 ∼350 mm^3^). Retreatment with a 2^nd^ cycle of sunitinib resulted in a rapid but transient tumor response (Fig. 4E, G). Growth of the NU12 tumors in mice treated with one cycle of [^177^Lu]Lu -cG250 RIT or one cycle of Su+[^177^Lu]Lu -cG250 RIT resumed 9-10 weeks after termination of therapy and tumors grew steadily thereafter (Fig. 4A,C,G,H). In contrast, when mice were treated with two cycles of [^177^Lu]Lu -cG250 RIT or two cycles of Su+[^177^Lu]Lu -cG250 RIT, complete tumor regression was observed, which continued until the end of the experiment. Statistical analysis showed statistically significant differences between the various treatment groups (p<0.001; day 91). At the end of the experiment (day 147), all animals treated with two cycles of Su+[^177^Lu]Lu -cG250 RIT or two cycles of [^177^Lu]Lu -cG250 RIT survived, (Fig. 4I and Table S1) and >80% of animals were tumor free. Seventy one percent of mice survived when treated with one cycle of [^177^Lu]Lu -cG250 RIT, but <30% of animals were tumor free. Treatment of any of the other treatments was inferior.

**Table 3:** Median tumor volumes of NU12.

	Day														
	14			42			55			91			160		
	mean	95% confidence interval		Mean	95% confidence interval		mean	95% confidence interval		mean	95% confidence interval		mean	95% confidence interval	
Control	**261**	172	396	**1456**	801	2646	**2857**	1420	5747						
Su 2x	**63**	43	95	**352**	200	620	**489**	259	924	**1253**	464	3388	**4371**	323	59090
Lu-cG250 1x	**72**	48	108	**63**	36	111	**49**	26	92	**34**	13	87	**56**	10	319
Lu-cG250 2x	**88**	60	132	**80**	45	140	**62**	33	117	**25**	10	64	**3**	1	17
Su + Lu-cG250 1x	**46**	31	69	**55**	31	98	**51**	27	97	**48**	18	128	**234**	38	1437
Su + Lu-cG250 2x	**59**	40	88	**70**	40	123	**60**	32	114	**28**	11	73	**2**	0.3	11

Median tumor volumes of NU12 tumors by treatment group and follow up day with uncorrected 95% confidence limits. Mixed model analysis was used for comparing the tumor growth of the six treatment groups. Using this model, the median tumor volume at days 14 (end 1^st^ cycle of Sunitinib (Su), 42 (start 2^nd^ cycle), 55 (end 2^nd^ cycle of Su), 91 (evaluation 2^nd^ cycle) and 147 was compared among treatment groups for selected hypotheses. Correction for multiple comparisons was done per day using Holm's method (improved Bonferroni method). Vol: median tumor volume, 1x: one cycle, 2x: two cycles. *** p<0.001 vs control. ^•••^ p < 0.001 vs sunitinib 2x °°° p<0.001 vs Su+[^177^Lu]Lu-cG250 RIT 1x. (for calculated p-values see Table S3).

Based on morphology by HE and Ki67 expression, 83% and 86% of the mice treated with two cycles of Su+Lu-cG250 RIT or two cycles of [^177^Lu]Lu -cG250 RIT were cured: no viable tumor cells were detected (data not shown). One cycle of [^177^Lu]Lu -cG250 RIT resulted in 29% of cured mice (no evidence of disease) and 14% and 8% of mice were completely tumor-free when treated with two cycles of Su or one cycle of Su+[^177^Lu]Lu -cG250 RIT respectively (Table S1).

## Discussion

To improve the therapy outcome of patients with mRCC new approaches are urgently needed.

In previous studies we found that the RCC models used here, SK-RC-52 and NU12, differ in their intrinsic response to sunitinib [8]. In the current study we examined inherent differences between the cell lines as well as microenvironmental factors to explain the observed difference in sensitivity to sunitinib and performed combination therapy studies to examine whether despite different sunitinib sensitivity, tumor cure could be achieved.

In vitro, both cell lines exhibited similar sunitinib sensitivity, and both were more sensitive for the active metabolite. However, the N-desethyl sunitinib IC_50_ of NU12 was substantially higher, suggesting a more sunitinib resistant phenotype. Long-term sunitinib treatment of NU12 led to an increased proliferation rate, as demonstrated by the decreased doubling time and conversion to a mesenchymal morphology, suggesting a more aggressive phenotype. In contrast, long-term sunitinib treatment of SK-RC-52 did not lead to changes in proliferation rate or morphological changes.

Continuous sunitinib exposure of NU12 enhanced pMET and pAXL expression and in SK-RC-52 SuR cells, (p)MET and (p)AXL changes were minimal and suggested MET and AXL activation. This suggested that SK-RC-52 cells are less sunitinib-sensitive as an alternative signaling pathway is constitutively active, however, IC_50_ values of untreated cells indicated that the cell lines did not differ in sunitinib sensitivity.

Although in vitro experiments showed that both cell lines were equally sunitinib sensitive, NU12 tumors responded significantly better albeit that long-lasting responses were not observed. This suggests that the NU12 tumor vasculature is more sunitinib sensitive, whereas the SK-RC-52 vasculature can resist this challenge. This is in agreement with previous studies showing that NU12 tumors were more sensitive to sunitinib than SK-RC-52 tumors: treatment with sunitinib leads to massive necrosis and vascular ablation and NU12 tumor regression whereas SK-RC-52 tumors stabilized without substantial necrosis and vascular changes [8]. Clearly, the differential sunitinib response cannot be attributed to inherent differences between NU12 and SK-RC-52.

NGS was performed to investigate whether the different response was related to differences in VEGF and PDGF expression by the tumor cells and corresponding receptor levels in the vascular bed [14]. NU12 and SK-RC-52 differed substantially in their VEGF/PDGF expression profile: SK-RC-52 showed much higher VEGFC levels and lower VEGFD expression levels. In contrast, VEGF-A, VEGF-B, VEGF-D and PGF expression levels were significantly higher in NU12. Also, the VEGFR expression differed between the two models: VEGFR1-2, expression levels were much higher in the vascular bed of NU12 tumors and expression of VEGFR3, the receptor of VEGFC, the principal inducer of lymphangiogenesis [14,15], also promoting vascular permeability, was completely absent from the vascular bed of SK-RC-52 tumors. Similar results were obtained by RT2 Profiler™ PCR Array Human Angiogenesis (results not shown). Park et al. showed that the combination of high PGF and high VEGFA expression levels might play a major role in tumor angiogenesis [16]. PGF selectively binds to VEGFR1 leading to even more VEGFR signaling in NU12. The collective results may explain the difference between the models toward sunitinib treatment: in NU12 tumors vascularization is mainly driven by VEGFA/B but also by PGF and the tumor vasculature highly expresses sunitinib's main targets VEGFR1,2,3 facilitating destruction of endothelial cells by sunitinib. In contrast, in SK-RC-52 tumors, VEGFR1 expression level is substantially lower, and VEGFC expression levels substantially higher. Although VEGFC, an essential chemotactic and survival factor during lymphangiogenesis is higher expressed, VEGFR3, the major ligand for VEGFC, was not expressed in the endothelial cells of these tumors, suggesting that lymphangiogenesis is less prominent [17]. Based on the VEGFR expression levels it appears that SK-RC-52 tumors mainly signal through VEGFR2, driven by VEGFA/B and VEGFC [15]. The higher VEGFC expression levels may lead to enhanced vascular permeability through VEGFR2 binding [18,19]. Moreover, the lower PDGFRB expression level also indicates less stable and more permeable vasculature in SK-RC-52. The difference in VEGF/VEGFR phenotype fits well with previous experiments where we showed that microvessel density (MVD) was much higher in NU12 tumors compared to SK-RC-52 tumors i.e., 20% and ∼1% respectively. The VEGF and VEGFR expression differences are also in line with the observed dissimilarity in sunitinib tumor responses: treatment leads to major necrosis in NU12 tumors (high expression VEGFR1) and stabilization of SK-RC-52 tumors (lower VEGFR1) [8].

The results also shed light on the antibody accumulation patterns that we previously observed with enhanced tumor-specific antibody accumulation in SK-RC-52 tumors after sunitinib treatment. Quite likely the vascular bed of SK-RC-52 tumors is insufficiently affected by sunitinib treatment to result in ablation of the vascular bed, but the damage may lead to enhancement of the vascular permeability and therefore higher antibody accumulation. In contrast, in NU12 tumors the vasculature is completely destroyed blocking antibody-accumulation through blood flow [20].

In view of the observed differences, supporting that they represent different, clinically relevant phenotypes, we performed therapy experiments in the two RCC models and combined sunitinib therapy with cG250-RIT since a concerted attack on the tumor vasculature and tumor cells may be more efficacious. We restricted the sunitinib treatment to relatively short periods to avoid the occurrence and influence of sunitinib resistance and to mimic treatment cycles in patients, which could influence the outcome of the studies. Treatment with sunitinib alone was insufficient to cure the animals in either model. When we combined sunitinib with CAIX-targeted RIT, aimed at the vascular bed (sunitinib) and tumor cells ([^177^Lu]Lu-cG250), overall survival increased, but ultimately tumors recurred in most mice. Addition of a second combination treatment cycle resulted in survival in most animals in both models. Apparently, this combination treatment can overcome the sunitinib resistance of SK-RC-52 tumors. In the SK-RC-52 model the superior effect of the combination treatment is likely a reflection of the sunitinib-induced enhanced antibody accumulation, leading to increased tumor radiation doses. Nevertheless, it must be noted that most animals were not completely tumor free, indicating that more treatment cycles are necessary to achieve complete cure. In NU12 tumors, sunitinib treatment leads to vascular collapse and extensive central necrosis and the remaining viable tumor cells surrounding the necrotic core can be ablated by the targeted RIT. NU12 tumors are quite cG250-RIT sensitive, as shown by the effects of two [^177^Lu]Lu-cG250 RIT cycles alone and as such these treatments are interchangeable.

Collectively our results suggest that (part of) the sunitinib resistance observed in mRCC patients might be explained by difference in the permeable status of ccRCC tumors, of which the models used here are examples. With high VEGFR1,2,3 expression, high PGF, VEGFA, VEGF-B and VEGF-D levels (and subsequently High MVD) and absence of pMET and pAXL NU12 can be seen as a classic sunitinib sensitive tumor, whereas SK-RC-52 tumors with no VEGFR3 expression, high VEGFC levels and the possibility to use redundant proliferation pathways are less dependent on the angiogenic component and represent a more sunitinib-resistant phenotype.

Combination treatment with [^177^Lu]Lu-cG250 RIT was successful in SK-RC-52, the sunitinib-resistant model, emphasizing that targeting blood vessels and tumor cells is more effective than targeting either compartment alone. The combination treatment was superior compared to two cycles of sunitinib and to [^177^Lu]Lu -cG250 RIT alone. This suggests that this combination treatment might be a possible option for mRCC patients ineligible or unable to receive CI therapy. Several studies have confirmed the feasibility and enhanced efficacy of combination therapy of antibody and TKI [21,22]. Kelly et al. [23] observed that combination of [^177^Lu]Lu -hu3S193 RIT with EGFR inhibitor AG1478 significantly improved efficacy in mice with prostate carcinoma. The enhanced effect with the EGFR inhibitor was attributed to the simultaneous targeting of tumor cells by two different drugs. In a recently performed meta-analysis for the treatment of non-small cell lung cancer, the authors showed that chemotherapy or EGFR-TKIs with bevacizumab significantly prolonged PFS and OS as first-line treatment for NSCLC compared with chemotherapy or TKIs alone, indicating that combinations can be more efficacious [24]. Here we combined tumor-specific antibody with TKI, targeting two different tumor components (tumor cells and vasculature) and demonstrated substantial improvement of efficacy. Whether this combination is superior to simultaneous targeting of tumor cells with different drugs remains to be established.

Although sunitinib alone showed little SK-RC-52 tumor growth inhibition, an additive effect was observed when combined with [^177^Lu]Lu-cG250 RIT, possibly due to the enhanced uptake of G250 antibody [8]. Recently, Jedeszko et al. [25] investigated the combination of pazopanib and chemotherapy in an orthotopic RCC mouse model and claimed that pazopanib enhanced the intracellular uptake of a chemotherapeutic drug by a direct sensitization effect on tumor cells. It is possible that a similar effect applies to our studies and that sunitinib has a direct sensitization effect on SK-RC-52 tumor cells, leading to increased uptake of mAb G250. The enhanced uptake was not observed in NU-12 tumors, but this can be explained by different internalization rates of the two tumor cell types [26]. In SK-RC-52, cG250 internalization and subsequent metabolization plays a role whereas internalization is almost absent in NU-12.

A limitation of this study is that although the combination treatment was successful in tumor ablation, the CAIX-targeted RIT was still too effective to objectively measure the additive effect of sunitinib in NU12. Additional therapeutic experiments with even lower doses of ^177^Lu should be performed to confirm the superiority of the combination treatment. Nevertheless, it also suggests that much lower [^177^Lu]Lu -cG250 doses might be effective, which will prevent bone marrow toxicity, the main dose limiting toxicity for RIT.

## Conclusions

In conclusion, since the efficacy of targeted agents such as TKI is limited by both intrinsic and acquired resistance for patients with advanced, improvement is needed. Enhanced therapeutic efficacy was achieved when sunitinib and [^177^Lu]Lu-cG250 RIT, two agents that on their own do not induce satisfactory response levels in preclinical models, are combined. Our findings provide a promising new therapeutic strategy for patients with advanced RCC.

### Institutional review board statement

Institutional guidelines were strictly followed for maintenance of animals and experimental procedures were approved by the Institutional Animal Care and Use Committee (IACUC, RU-DEC 2012-038 and RU-DEC 2012-267). All procedures were performed using the guidelines from the Institute of Laboratory Animal Research [32].

## Author contributions

Conceptualization J.O-W., E.O., P.M.; methodology J.O-W., E.O., G.F., M.dW., O.B.; formal analysis T.dH., D.K; investigation J.O-W., M.dW., G.F.; resources J.O-W, G.F., M.dW; data curation J.O-W; writing—original draft preparation J.O-W., E.O.; writing—review and editing J.O-W., E.O., M.dW., G.F., O.B., P.M.; visualization J.O-W., M.dW., T.dH.; supervision J.O-W., E.O., P.M.; project administration J.O-W.; funding acquisition E.O., P.M. All authors have read and agreed to the published version of the manuscript.

## Funding

This work has been supported in part by the European Union's Seventh Framework Program (FP7/2007-2013) under grant agreement no 259939 www.eurotargetproject.eu.

## Data availability statement

The dataset used in this study is available in EGA (https://ega-archive.org) with access number EGAD00001008312.

## Appendix Supplementary materials

Image, application 1
