# Supplementary material for: Combination of sunitinib and 177Lu-labeled antibody cG250 targeted radioimmunotherapy: A promising new therapeutic strategy for patients with advanced renal cell cancer
Source: Neoplasia. 2022 Jul 22;32:100826. doi: 10.1016/j.neo.2022.100826 (PMC9309230; doi:10.1016/j.neo.2022.100826)

**Supplementary Materials**

Figure S1: Treatment schedule of mice with SK-RC-52 or NU12 tumors, Table S1: Survival of mice with SK-RC-52 or NU12 tumors following treatment with sunitinib and/or [177Lu]Lu -cG250 RIT, Table S2: p-values for comparison of treatment groups of mice with SK-RC-52 tumors, Table S3: p-values for comparison of treatment groups of mice with NU12 tumors.


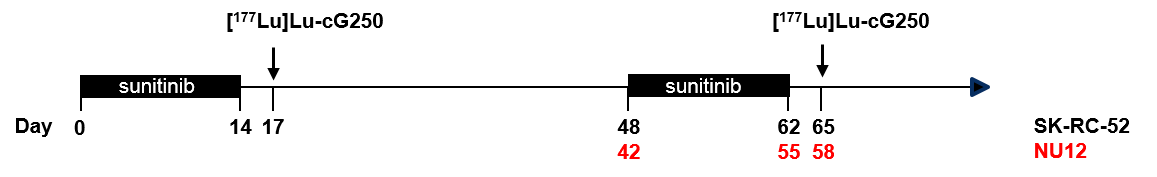

Supplement: Supplementary file 1 [file mmc1.zip › mmc1.docx]
