# Supplementary material for: Combination of sunitinib and 177Lu-labeled antibody cG250 targeted radioimmunotherapy: A promising new therapeutic strategy for patients with advanced renal cell cancer
Source: Neoplasia. 2022 Jul 22;32:100826. doi: 10.1016/j.neo.2022.100826 (PMC9309230; doi:10.1016/j.neo.2022.100826)
Supplement: Supplementary file 1 [file mmc1.zip › Table S2 statistics SK-RC-52.docx]

**Table S2. p-values for comparison of treatment groups of mice with SK-RC-52 tumors**

| **Day** | **Group** | **[177Lu]Lu-cG250 All groups** | **Su**  **All groups** | **Su + [177Lu]Lu-cG250**  **All groups** | **Su**  **2x** | **[177Lu]Lu-cG250 1x** | **[177Lu]Lu-cG250 2x** | **Su + [177Lu]Lu-cG250**  **1x** | **Su + [177Lu]Lu-cG250**  **2x** |
| --- | --- | --- | --- | --- | --- | --- | --- | --- | --- |
|  |  | Adjusted P-value | Adjusted P-value | Adjusted P-value | Adjusted P-value | Adjusted P-value | Adjusted P-value | Adjusted P-value | Adjusted P-value |
| 14 | Control | 0.828 | 0.168 |  |  |  |  |  |  |
|  | Su, all groups | 0.043 |  |  |  |  |  |  |  |
| 48 | Control | 0.126 |  | <.001 | 0.442 |  |  |  |  |
| 62 | Control |  |  |  | 0.444 |  |  |  |  |
| 98 | [177Lu]Lu-cG250 2x |  |  |  |  |  |  |  | 0.123 |
|  | Control |  |  |  | 1.000 | 1.000 | 0.123 | 0.017 | <.001 |
| 160 | [177Lu]Lu-cG250 2x |  |  |  |  |  |  |  | 0.121 |

The median tumor volume of mice with SK-RC-52 tumors was compared at days 14 (end 1^st^ cycle of sunitinib (Su), 48 (start 2^nd^ cycle), 62 (end 2^nd^ cycle of Su), 98 (evaluation 2^nd^ cycle) and 160 among treatment groups for selected hypotheses. For each treatment group the development in time was described with a fifth-degree polynomial (fixed effects). Individual mice were allowed to follow their own curve (all six coefficients for the polynomial function in time were random). Using this model, the median tumor volume at days 14 (end 1st cycle of sunitinib (Su), 48 (start 2nd cycle), 62 (end 2nd cycle of Su), 98 (evaluation 2nd cycle) and 160 was compared among treatment groups for selected hypotheses. Correction for multiple comparisons was done per day using Holm’s method (improved Bonferroni method).
