# Supplementary figures and images for: Combination of sunitinib and 177Lu-labeled antibody cG250 targeted radioimmunotherapy: A promising new therapeutic strategy for patients with advanced renal cell cancer
Source: Neoplasia. 2022 Jul 22;32:100826. doi: 10.1016/j.neo.2022.100826 (PMC9309230; doi:10.1016/j.neo.2022.100826)

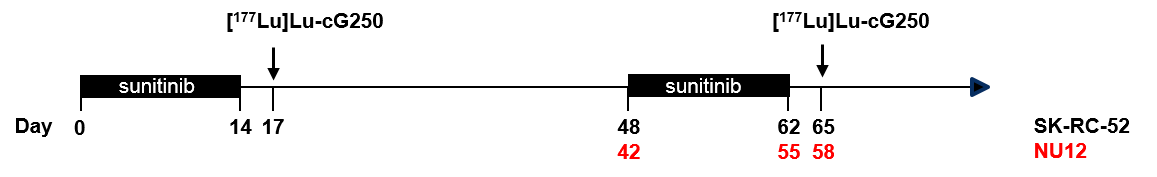

Supplement: Supplementary file 1 [file mmc1.zip › Figure S1.tif]
